# Impact of Chronic Kidney Disease on Clinical Outcomes during Hospitalization and Five-Year Follow-Up after Coronary Artery Bypass Grafting

**DOI:** 10.1155/2023/9364913

**Published:** 2023-09-26

**Authors:** Mohamed Laimoud, Mosleh Nazzel Alanazi, Mary Jane Maghirang, Shatha Mohamed Al-Mutlaq, Suha Althibait, Rasha Ghamry, Rehan Qureshi, Boshra Alanazi, Munirah Alomran, Zeina Bakheet, Zohair Al-Halees

**Affiliations:** ^1^Cardiovascular Critical Care Department, King Faisal Specialist Hospital and Research Center, Riyadh, Saudi Arabia; ^2^Critical Care Medicine Department, Cairo University, Cairo, Egypt; ^3^Cardiovascular Nursing Department, King Faisal Specialist Hospital and Research Center, Riyadh, Saudi Arabia; ^4^Cardiac Surgery Department, King Faisal Specialist Hospital and Research Center, Riyadh, Saudi Arabia; ^5^Nephrology Department, King Faisal Specialist Hospital and Research Center, Riyadh, Saudi Arabia; ^6^College of Medicine, Almaarefa University, Riyadh, Saudi Arabia; ^7^College of Medicine, Alfaisal University, Riyadh, Saudi Arabia

## Abstract

**Background:**

Chronic kidney disease (CKD) is often associated with multiple comorbidities including diabetes mellitus, and each has its own complications and impact after cardiac surgery including coronary revascularization. The objective of this work was to study the impact of CKD on clinical outcomes after coronary artery bypass grafting (CABG) and to compare outcomes in patients with different grades of renal functions. We retrospectively reviewed all patients who underwent CABG from January 2016 to August 2020 at our tertiary care hospital using electronic medical records.

**Results:**

The study included 410 patients with a median age of 60 years, and 28.6% of them had CKD and hospital mortality of 2.7%. About 71.4% of the patients had GFR > 60 mL/min per 1.73 m^2^, 18.1% had early CKD (GFR 30–60), 2.7% had late CKD (GFR < 30), and 7.8% of them had end-stage renal disease (ESRD) requiring dialysis. The CKD group had significantly more frequent hospital mortality (*p* = 0.04), acute cerebrovascular stroke (*p* = 0.03), acute kidney injury (AKI) (*p* < 0.001), longer ICU stay (*p* = 0.002), post-ICU stay (*p* = 0.001), and sternotomy wound debridement (*p* = 0.03) compared to the non-CKD group. The frequencies of new need for dialysis were 2.4% vs. 14.9% vs. 45.5% (*p* < 0.001) in the patients with GFR > 60 mL/min per 1.73 m^2^, early CKD, and late CKD, respectively. Acute cerebral stroke (OR: 10.29, 95% CI: 1.82–58.08, and *p* = 0.008), new need for dialysis (OR: 25.617, 95% CI: 13.78–85.47, and *p* < 0.001), and emergency surgery (OR: 3.1, 95% CI: 1.82–12.37, and *p* = 0.036) were the independent predictors of hospital mortality after CABG. The patients with CKD had an increased risk of strokes (HR: 2.14, 95% CI: 1.20–3.81, and *p* = 0.01) but insignificant mortality increase (HR: 1.44, 95% CI: 0.42–4.92, and *p* = 0.56) during follow-up.

**Conclusion:**

The patients with CKD, especially the late grade, had worse postoperative early and late outcomes compared to non-CKD patients after CABG. Patients with dialysis-independent CKD had increased risks of needing dialysis, hospital mortality, and permanent dialysis after CABG.

## 1. Background

Coronary artery bypass grafting (CABG) is surgical coronary revascularization in patients with advanced coronary artery disease who are unsuitable for or after unsuccessful trials of percutaneous coronary interventions (PCI) [1]. Chronic kidney disease (CKD) is often associated with multiple comorbidities including diabetes mellitus, and each has its impact after cardiac surgeries and revascularization. There is a proven association between CKD and a high prevalence of cardiovascular disorders. Moreover, patients with end-stage renal disease (ESRD) have a 10–30-fold increased mortality compared with general people [2]. Chronic renal failure was linked to cardiovascular mortality due to myocardial dysfunction, systemic hypertension, chronic anemia, dyslipidemia, hyperhomocysteinemia and hyperfibrinogenemia, nitric oxide/endothelin imbalance, oxidative stress, and chronic inflammation [2–5]. Many studies proved the association between ESRD and different worse outcomes after CABG [6–8]. We conducted this study to identify the impact of CKD on outcomes after CABG and to compare the outcomes in patients with different grades of renal functions.

## 2. Methods

### 2.1. Study Design and Data Collection

We conducted this retrospective study including all adult patients who underwent CABG between 2016 and 2020 in our tertiary care hospital. We collected the variables studied from electronic hospital records. The study was approved by the Ethical Committee of King Faisal Specialist Hospital and Research Center and waived from specific consent as there were no personally identifiable data or photos. The study reference number is 2211015, and the publication number is 22350215021. The data collected included demographic and perioperative variables. The primary outcome was hospital mortality, while the secondary outcomes included cerebrovascular stroke, new need for dialysis, sternotomy wound debridement, and length of hospitalization. The studied variables included patients' characteristics, risk factors of cardiovascular and cerebrovascular diseases, prior revascularization, left ventricular ejection fraction, perioperative mechanical circulatory support, and laboratory workup including blood lactate, troponin, and glycated hemoglobin (HbA1c). The operative data collected included cardiopulmonary and aortic cross-clamping times, urgency and the approach of surgery, surgical bleeding, and need for exploration. The postoperative data collected included acute kidney injury (AKI), new need for dialysis, acute cerebrovascular stroke, mortality, length of stay, gastrointestinal bleeding, arrhythmias, sternotomy wound infection, and need for debridement. Follow-up data included mortality and acute cerebrovascular stroke.

According to the Kidney Disease: Improving Global Outcomes (KDIGO) clinical practice guidelines, CKD was defined as a glomerular filtration rate (GFR) less than 60 mL/min/1.73 m^2^ for ≥3 months irrespective of etiology [9, 10]. According to the degree of kidney injury, there were 5 stages of CKD. Because of imprecision to accurately estimate GFR at higher ranges, it is difficult to distinguish between stages 1 and 2. Clinically, CKD was classified according to severity into early CKD, late CKD, and ESRD [9] (Table 1). AKI was defined as an acute reduction of renal functions as monitored by urine output or GFR according to the risk, injury, failure, loss, and end-stage renal disease (RIFLE) criteria [11].

### 2.2. Statistical Analysis

Data were summarized using the median with an interquartile range in quantitative data and frequency (count) with relative frequency (percentage) for categorical data. The nonparametric Kruskal–Wallis and Mann–Whitney tests were used for quantitative variable comparison. The Chi-square (c2) test was performed to compare the categorical variables. Two-sided *p* values were considered statistically significant if < 0.05. Multivariate logistic regression was performed to get the predictors of hospital mortality and acute cerebrovascular stroke. We obtained the Kaplan–Meier survival curves using the log-rank test. The Hosmer–Lemeshow test was used to detect the goodness of fit of the regression models. Variance inflation testing was performed to detect multicollinearity of the regression models. The Statistical Package for Social Sciences (SPSS) version 26 (IBM Corp., Armonk, NY, USA) was used in our study analysis.

## 3. Results

### 3.1. Baseline Clinical Variables of the Studied Patients

Four hundred and ten adult patients with a median age of 60 (55–68) years were enrolled in our study and divided into 2 groups according to presence of CKD. A total of 117 (28.6%) patients had CKD, while 293 (71.4%) patients did not have CKD before CABG (Table 2).

According to the glomerular filtration rate (GFR), the studied patients were subdivided into 4 groups; 292 (71.2%) patients had GFR > 60 mL/min/1.73 m^2^, 74 (18.05%) patients had early CKD (GFR 30–60 mL/min/1.73 m^2^), 11 (2.7%) patients had late CKD (GFR less than 30 mL/min/1.73 m^2^), and 32 (7.8%) patients had end-stage renal disease (ESRD) requiring dialysis (Table 3).

### 3.2. Laboratory Variables of the Studied Patients

Preoperatively, the studied patients with CKD had significantly lower median levels of hemoglobin (*p* < 0.001), albumin (*p* < 0.001), and bilirubin (*p* < 0.001) but a higher median HBA1c level (*p* = 0.003) compared to the patients without CKD, respectively. Postoperatively, the patients with CKD had significantly higher blood lactate (*p* = 0.006) and troponin levels (*p* < 0.001) compared to the non-CKD group, respectively (Table 2).

### 3.3. Operative Details and Outcomes of the Studied Patients

The operative details and use of mechanical circulatory support were statistically insignificant among the studied patients with different grades of GFR. The frequencies of new need for dialysis were 2.4% vs. 14.9% vs. 45.5% (*p* < 0.001) in the patients with GFR > 60 mL/min per 1.73 m^2^, early CKD, and late CKD, respectively. The CKD group had significantly more frequent acute ischemic cerebrovascular stroke (*p* = 0.03), higher hospital mortality (*p* = 0.04), longer ICU (*p* = 0.002), and post-ICU stay (*p* = 0.001) than the non-CKD group (Tables 4 and 5).

**Figure 1:**
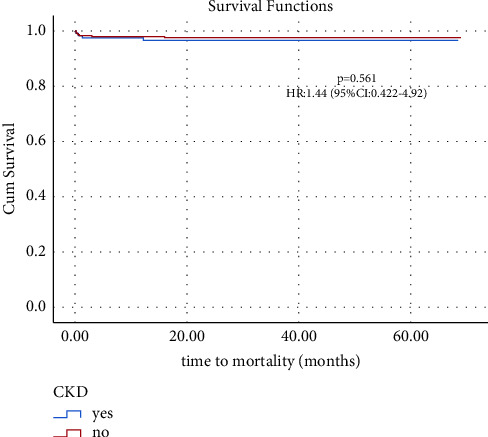
Kaplan–Meier curves of survival for 5-year follow-up.

**Figure 2:**
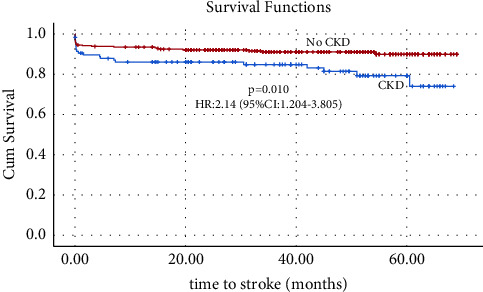
Kaplan–Meier curves of cerebrovascular stroke for 5-year follow-up.

Kaplan–Meier curves revealed that the CKD group had an increased risk of cerebrovascular strokes (HR: 2.14, 95% CI: 1.20–3.81, and *p* = 0.01) but insignificant mortality increase (HR: 1.44, 95% CI: 0.42–4.92, and *p* = 0.56) compared to the non-CKD group (Figures 1 and 2).

### 3.4. Predictors of Hospital Mortality and Cerebrovascular Stroke

Acute postoperative stroke (OR = 10.29, 95% CI: 1.823–58.08, and *p* = 0.008), new need for dialysis (OR = 25.617, 95% CI: 13.78–85.47, and *p* < 0.001), and emergency surgery (OR = 3.1, 95% CI: 1.82–12.37, and *p* = 0.036) were the independent predictors of hospital mortality after CABG in our logistic regression analysis. Compared to survivors, nonsurvivors had significantly more frequent CKD, prior cardiotomies, postoperative atrial fibrillation, exploration because of mediastinal bleeding, and CABG plus valve surgeries with higher peak levels of troponin and blood lactate levels, but these variables were insignificant in the logistic regression analysis. The regression model had a goodness of fit by the Hosmer–Lemeshow test (Pearson chi^2^ = 57.61, *p* value = 1), and the mean variance inflation factor was 1.19 (Tables 6 and 7).

Postoperative cerebrovascular stroke was independently predicted with carotid artery stenosis (OR: 9.38, 95% CI: 3.7–23.7, and *p* < 0.001), atrial fibrillation (OR: 2.35, 95% CI: 1.013–5.4, and *p* = 0.016), CABG plus valve surgery (OR: 7.44, 95% CI: 2.75–20.15, and *p* < 0.001), HBA1c (OR: 1.43, 95% CI: 1.07–1.91, and *p* = 0.016), CKD (OR: 2.33, 95% CI: 1.06–5.17, and *p* = 0.036), and preoperative history of stroke (OR: 3.73, 95% CI: 1.18–11.77, and *p* = 0.025) in the logistic regression analysis. The regression model had a goodness of fit by the Hosmer–Lemeshow test (Pearson chi^2^ = 38.37, *p* value = 0.64), and the mean variance inflation factor was 1.15 (Table 7).

## 4. Discussion

Our study showed that the presence of CKD before CABG was associated with increased hospital mortality, multiple morbidities, and prolonged hospitalization. The overall mortality in this cohort was 2.7%, and it ranged from 2.1% in patients with GFR >60 mL/min/1.73 m^2^ to 9.1% in patients with late CKD. Our mortality report was similar to Cooper et al.'s study [12] that reported a hospital mortality of 2.5%, and it was 9.3% in patients with late CKD and 9% in hemodialysis dependents before CABG. Reddan et al. [13] studied the relation between GFR and hospital mortality after revascularization and documented that each GFR decline of 10 ml/min/1.73 m^2^ was associated with a 14% increased risk of mortality, but at GFR >85 mL/min/1.73 m^2^, the relation was attenuated. The subgroup analysis revealed a lower mortality in preoperative dialysis-dependent patients compared to the late group which may be related to the small group size. Moreover, in our cohort, 5% of the late CKD group required dialysis during hospitalization, and the new need for dialysis was a predictor of hospital mortality. Liu et al. [6] reported that preoperative hemodialysis had a three-fold increase in hospital mortality after CABG. Yamauchi et al. [7] reported significantly higher operative and 30-day mortality in hemodialysis compared to nonhemodialysis patients after CABG. Safaie et al. [8] reported a hospital mortality of 10.5% in CKD patients who underwent CABG, and there was an insignificant difference between dialysis-dependent and nondialysis-dependent patients.

Acute cerebrovascular stroke occurred in 7.3% of the patients after CABG in our study. Compared to the non-CKD group, the patients with CKD, especially those with late CKD, had an increased risk of acute stroke during hospitalization and the 5-year follow-up. Moreover, preoperative CKD was a predictor of acute postoperative stroke in the logistic regression analysis. Cooper et al. [12] reported that late CKD and dialysis dependence were associated with postoperative stroke. Liu et al. [6] reported that patients with preoperative hemodialysis dependence were 2.1 times more likely to have postoperative stroke. Preoperative CKD carried an increased risk of acute cerebrovascular stroke during the follow-up after CABG [5, 14].

Regarding other postoperative morbidities, the study results revealed a higher frequency of sternotomy wound debridement in the CKD group compared to the non-CKD group. There was a statistically insignificant difference between both groups regarding mediastinal exploration for bleeding. The CKD group had longer hospitalizations with more days of vasopressor and IABP support compared to the non-CKD group. These findings were similar to those of Cooper et al.'s report [12] which revealed prolonged hospitalization, deep sternal wound infection, and exploration for bleeding, especially in late CKD and dialysis-dependent patients. Liu et al. [6] reported that preoperative hemodialysis dependence carried a higher risk of mediastinitis but an insignificant difference for exploration because of bleeding compared to nondialysis dependence. CKD was linked to postcardiotomy 30-day and 1-year mortality [15, 16]. However, Powell et al. [17] conducted a small study and reported that dialysis dependence was associated with prolonged hospitalization without an increase in perioperative mortality and morbidities. Kan and Yang [18] reported that uremia was associated with bleeding tendency, prolonged ICU stay, and late mortality after CABG.

The patients with CKD have abnormal hemostatic profiles with abnormal risks of bleeding and thrombotic events. Ocak et al. [19] studied 10,347 patients and reported that patients with GFR <45 mL/min per 1.73 m^2^ and albuminuria had a 3.5-fold increased risk of bleeding. Platelet dysfunction was described in patients with advanced renal impairment as uremic thrombocytopathy and was related to decreased thromboxane A2 formation and von Willebrand factor (vWF) defect [20]. Renal impairment has been included in multiple bleeding risk scores such as ATRIA and HAS-BLED scores [21, 22]. The abnormal hemostatic profiles of CKD with added effects of cardiopulmonary bypass may lead to increased postoperative bleeding and thromboembolic complications. Cardiopulmonary bypass circulation leads to acute phase reaction and systemic inflammatory response with platelet activation and consumption and possible thromboembolic events [23]. The enrolled patients in this study underwent on-pump CABG, and there were insignificant differences regarding cardiopulmonary bypass and aortic clamping times in the CKD and non-CKD groups. It is still controversial to select on-pump or off-pump during CABG for better postoperative outcomes. Lamy et al. [24] reported the advantages of off-pump CABG regarding lower rates of AKI and respiratory and bleeding complications but similar rates of 30-day mortality, cerebrovascular stroke, myocardial infarction, and new need for dialysis compared to on-pump CABG. Li et al. [25] reported higher 30-day mortality and worse outcomes including new need for dialysis in the CKD group after off-pump CABG compared to the non-CKD group. Ueki et al. [26] reported that off-pump CABG significantly decreased the postoperative mortality in patients with CKD and decreased the need for dialysis in patients with late CKD.

In our study, deep sternal wound infection that required debridement was significantly more frequent in the CKD group compared to the non-CKD group. Our findings were consistent with Cooper et al.'s report [12]. Ishigami et al. [27] studied 9,697 patients with a 13.6-year median follow-up and reported increased hazard ratios (HRs) of infection-related hospitalizations and death with the decline of GFR. CKD is associated with a chronic low-grade inflammatory state with increased proinflammatory cytokines, resulting in increased cardiovascular risks, infections, and malignancy [28, 29].

CKD is associated with increased cardiovascular and cerebrovascular insults due to accelerated atherosclerosis, chronic inflammation, vascular calcification, electrolytes abnormalities, and anemia in addition to associated comorbidities including diabetes mellitus, dyslipidemia, and hypertension. Vascular calcification affects both the intima and media of arteries causing stiffness which increase the systolic and decrease diastolic blood pressure, resulting in decreased coronary perfusion, increased left ventricular afterload, and increased cardiovascular mortality [5, 29]. Calcification of the vascular media is the characteristic of CKD patients, described as Monckebergʼs sclerosis, and results from hyperphosphatemia [30]. Impaired renal phosphate secretion results in hyperphosphatemia and increased cardiovascular mortality [5]. CKD is associated with anemia due to decreased erythropoietin secretion and results in decreased oxygen supply, increasing cardiovascular risks [31].

Another challenging point in management of patients with CKD is postoperative analgesia. Postoperative sternotomy pain requires strong analgesia to control the sympathetic stimulation and cardiovascular side effects. However, impaired pharmacokinetics of opiates in the presence of CKD can result in side effects including prolonged sedation, seizures, delirium, nausea, vomiting, and respiratory complications [32]. Appropriate postcardiac surgery analgesia is critical, especially in CKD, to achieve a balance of pain control with patient satisfaction and avoid drug accumulation with side effects [33, 34].

Finally, with the advances of medical care, the patients presented to CABG have advanced age and multiple comorbidities including CKD and dialysis dependence. Meticulous perioperative management including hemodynamics optimization, appropriate fluid and electrolyte management, and appropriate analgesia should be addressed to high-risk patients with CKD to minimize perioperative morbidities and mortality.

## 5. Conclusion

The patients with CKD, especially the late grade, had worse postoperative early and late outcomes compared to non-CKD patients after CABG. Patients with dialysis-independent CKD had increased risks of needing dialysis hospital mortality and permanent dialysis after CABG.

## 6. Limitations

This work was a single-center retrospective study with a relatively small sample size. It missed perioperative blood sugar control and the amount of postoperative bleeding and blood transfusions. We used preoperative glycated hemoglobin as a marker of blood sugar control in the studied patients. We could not get data on blood glucose variability and control during hospitalization. We studied the impact of renal impairment on outcome, but the study missed some key details about dialysis modalities and efficiency and the laboratory finding of parathormone and electrolytes. The enrolled patients in this study underwent on-pump CABG as this is the standard approach in our hospital, and we do not know the validity of our result for off-pump CABG. We studied the surgical wound infection without other infections during hospitalization.

## Figures and Tables

**Table 1:** Classes of chronic kidney disease [9].

Stages	Description	Classification	
		GFR (mL/min/1.73 m^2^)	Terms
1	Kidney damage with normal GFR	≥90	Proteinuria, albuminuria
2	Kidney damage with a mild decrease of GFR	60–89	Proteinuria, albuminuria
3	Moderately reduced GFR	30–59	Early renal insufficiency
4	Severely reduced GFR	15–29	Late renal insufficiency, pre-ESRD
5	Kidney failure	<15	ESRD

ESRD: end-stage renal disease; GFR: glomerular filtration rate.

**Table 2:** Demographic and clinical variables of the patients studied.

Studied variables		All patients (*n* = 410)	Patients with CKD (*n* = 117, 28.6%)	Patients without CKD (*n* = 293, 71.4%)	*P* value
Age (years)		60 (55–68)	63 (58–71)	59 (54–66)	0.07
Gender, male (*n*, %)		337 (82.2)	87 (74.4)	250 (85.3)	0.09
BMI (kg/m^2^)		28.05 (25.8–31.8)	28.3 (25.8–32)	28 (25.9–31.5)	0.79
Smoking (*n*, %)		159 (38.8)	35 (29.9)	124 (42.3)	0.02
Diabetes mellitus (*n*, %)		338 (82.4)	111 (94.9)	227 (77.5)	<0.001
Hyperlipidemia (*n*, %)		245 (59.8)	77 (65.8)	168 (57.3)	0.11
Hypertension (*n*, %)		351 (85.6)	110 (94)	241 (82.3)	0.002
Bronchial asthma (*n*, %)		44 (10.7)	25 (21.4)	19 (6.5)	<0.001
Hypothyroidism (*n*, %)		42 (10.2)	11 (9.4)	31 (10.6)	0.72
Hyperthyroidism (*n*, %)		3 (0.7)	0 (0)	3 (1)	0.56
Preoperative AF (*n*, %)		26 (6.3)	9 (7.7)	17 (5.8)	0.47
Autoimmune disease (*n*, %)		4 (1)	2 (1.7)	2 (0.7)	0.32
Prior stroke (*n*, %)		28 (6.8)	7 (6)	21 (7.2)	0.66
CAS (*n*, %)		37 (9)	17 (14.5)	20 (6.8)	0.014
PVD (*n*, %)		44 (10.7)	20 (17.1)	24 (8.2)	0.009
Prior MI (*n*, %)		257 (62.7)	87 (74.4)	170 (58)	0.002
Prior PCI (*n*, %)		137 (33.4)	45 (38.5)	92 (31.4)	0.17
Prior cardiac surgeries (*n*, %)		27 (6.6)	12 (10.3)	15 (5.1)	0.58
Preoperative LVEF%	>55%	141 (34.4)	34 (29.1)	107 (36.5)	0.08
	45–55%	106 (25.9)	25 (21.4)	81 (27.6)	
	35–45	88 (21.5)	36 (30.8)	52 (17.7)	
	<35%	75 (18.3)	22 (18.8)	53 (18.1)	
Preoperative IABP (*n*, %)		15 (3.7)	2 (1.7)	13 (4.4)	0.25
Preoperative ECMO (*n*, %)		2 (0.5)	0 (0)	2 (0.7)	1
Preoperative hemoglobin (g/L)		131 (115–142)	117 (101–132)	134 (121–145)	<0.001
Preoperative platelets (10^9^/L)		246 (202–308)	263 (208–316)	241 (201–305)	0.06
Preoperative serum bilirubin (*μ*mol/L)		6.6 (4.6–11.1)	5 (3.7–8.4)	7.6 (5–12.5)	<0.001
Preoperative INR		1 (1-1.1)	1.1 (1-1.1)	1 (1-1.1)	0.107
Preoperative fibrinogen (g/L)		3.2 (2.85–3.65)	3.26 (3.1–3.78)	3.17 (2.84–3.52)	0.051
Preoperative serum albumin (g/L)		39.8 (36.7–42.4)	37.1 (32.7–40)	41 (38–42.6)	<0.001
HBA1c (%)		7.2 (6.4–8.2)	7.5 (6.8–8.4)	6.9 (6.3–8.1)	0.003
Peak lactate (mmol/L)		5.5 (3.8–8.9)	6.3 (3.9–10.8)	5.1 (3.7–8.4)	0.006
Peak troponin (ng/L)		634 (392–1180)	814 (490–1427)	579 (379–1090)	<0.001

BMI: body mass index; CAS: carotid artery stenosis; CKD: chronic kidney disease; PVD: peripheral vascular disease; MI: myocardial infarction; PCI: percutaneous coronary intervention; AF: atrial fibrillation; IABP: intra-aortic balloon pump; ECMO: extracorporeal membrane oxygenation; LVEF: left ventricle ejection fraction.

**Table 3:** Demographic and clinical variables of the patients according to GFR.

Studied variables		Patients with GFR > 60 (*n* = 292, 71.4%)	Patients with early CKD (*n* = 74, 18.1%)	Patients with late CKD (*n* = 11, 2.7%)	Patients on dialysis (*n* = 32, 7.8%)	*P* value
Age (years)		59 (54–66)	66 (59–74)	60 (59–64)	65 (55–71)	0.057
Gender, male (*n*, %)		249 (85.3)	53 (71.6)	9 (81.8)	26 (81.3)	0.04
BMI (kg/m^2^)		28 (25.75–31.5)	28.9 (27.4−31.8)	26.6 (24.9–33.5)	27.05 (24.5–32)	0.139
Smoking (*n*, %)		123 (42.1)	23 (31.1)	5 (45.5)	8 (25)	0.118
Diabetes mellitus (*n*, %)		227 (77.7)	72 (97.3)	11 (100)	28 (87.5)	<0.001
Hyperlipidemia (*n*, %)		167 (57.2)	51 (68.9)	5 (45.5)	22 (68.8)	0.108
Hypertension (*n*, %)		240 (82.2)	69 (93.2)	11 (100)	31 (96.9)	0.016
Bronchial asthma (*n*, %)		19 (6.5)	17 (23)	1 (9.1)	7 (21.9)	<0.001
Hypothyroidism (*n*, %)		31 (10.6)	9 (12.2)	1 (9.1)	1 (3.1)	0.55
Hyperthyroidism (*n*, %)		3 (1)	0 (0)	0 (0)	0 (0)	1
Preoperative AF (*n*, %)		17 (5.8)	6 (8.1)	1 (9.1)	2 (6.3)	0.7
Autoimmune disease (*n*, %)		2 (0.7)	2 (2.7)	0 (0)	0 (0)	0.49
Prior stroke (*n*, %)		20 (6.8)	5 (6.8)	2 (18.2)	1 (3.1)	0.049
CAS (*n*, %)		19 (6.5)	12 (16.2)	2 (18.2)	4 (12.5)	0.026
PVD (*n*, %)		23 (7.9)	12 (16.2)	0 (0)	9 (28.1)	0.002
Prior MI (*n*, %)		170 (58.2)	58 (78.4)	10 (90.9)	19 (59.4)	0.004
Prior PCI (*n*, %)		91 (31.3)	37 (50)	1 (9.1)	8 (25)	0.003
Prior cardiac surgeries		15 (5.1)	7 (9.5)	0 (0)	5 (15.6)	0.08
Preoperative LVEF%	>55%	106 (36.3)	22 (29.7)	3 (27.3)	10 (31.3)	0.33
	45–55%	81 (27.7)	17 (23)	2 (18.2)	6 (18.8)	
	35–45	52 (17.8)	20 (27)	4 (36.4)	12 (37.5)	
	<35%	53 (18.2)	15 (20.3)	3 (27.3)	4 (12.5)	
Preoperative IABP (*n*, %)		13 (4.5)	2 (2.7)	0 (0)	0 (0)	0.79
Preoperative ECMO (*n*, %)		2 (0.7)	0 (0)	0 (0)	0 (0)	1

BMI: body mass index; CAS: carotid artery stenosis; CKD: chronic kidney disease; PVD: peripheral vascular disease; MI: myocardial infarction; PCI: percutaneous coronary intervention; AF: atrial fibrillation; IABP: intra-aortic balloon pump; ECMO: extracorporeal membrane oxygenation; LVEF: left ventricle ejection fraction.

**Table 4:** Operative details and outcomes of the studied patients.

Studied variables		All patients	Patients with CKD	Patients without CKD	*P* value
Type of surgery	Isolated CABG (*n*, %)	370 (90.2)	106 (90.6)	264 (90.1)	0.87
	CABG plus valve surgery (*n*, %)	40 (9.8)	11 (9.4)	29 (9.9)	
Cardiopulmonary bypass (minutes)		100 (77–126)	98 (75–128)	100 (78–125)	0.96
Aortic cross-clamping (minutes)		58 (43–83)	50 (42–77.5)	63 (44–84)	0.11
Approach	Sternotomy (*n*, %)	398 (97.1)	112 (95.7)	286 (97.6)	0.33
	Minimally invasive	12 (2.9)	5 (4.3)	7 (2.4)	
Urgency of surgery	Elective (*n*, %)	397 (96.8)	114 (97.4)	283 (96.6)	0.76
	Emergent (*n*, %)	13 (3.2)	3 (2.6)	10 (3.4)	
Exploration for bleeding (*n*, %)		27 (6.6)	6 (5.1)	21 (7.2)	0.45
New onset AF (*n*, %)		109 (26.6)	31 (26.5)	78 (26.6)	0.9
Ventricular arrhythmias (*n*, %)		24 (5.9)	8 (6.8)	16 (5.5)	0.59
Acute kidney injury (*n*, %)		105 (25.6)	45 (38.5)	60 (20.5)	<0.001
New need for hemodialysis (*n*, %)		24 (5.9)	17 (14.5)	7 (2.4)	<0.001
Gastrointestinal bleeding (*n*, %)		20 (4.9)	9 (7.7)	11 (3.8)	0.09
Sternotomy wound infection (*n*, %)		42 (10.2)	11 (9.4)	31 (10.6)	0.9
Wound debridement (*n*, %)		18 (4.4)	7 (5.9)	11 (3.6)	0.031
ICU days		4 (3–6)	5 (3–7)	4 (3–5)	0.002
Inotropes days		2 (1-2)	2 (1–4)	1 (1-2)	<0.001
Ventilator days		1 (1-2)	1 (1-2)	1 (1-1)	0.019
Post-ICU ward days		5 (3–8)	6 (4–10)	4 (3–6)	0.001
Postoperative ECMO (*n*, %)		8 (2)	3 (2.6)	5 (1.7)	0.69
Postoperative IABP (*n*, %)		32 (7.8)	8 (6.8)	24 (8.2)	0.64
Tracheostomy (*n*, %)		12 (2.9)	6 (5.1)	6 (2)	0.11
Hospital mortality (*n*, %)		11 (2.7)	5 (4.3)	6 (2.04)	0.04
Acute ischemic stroke (*n*, %)		30 (7.3)	12 (10.3)	18 (6.14)	0.03
Intracranial bleeding (*n*, %)		1 (3.2)	0 (0)	1 (0.34)	1
Stroke during follow-up (*n*, %)		15 (3.7)	8 (6.8)	7 (2.4)	0.01
Mortality during follow-up (*n*, %)		28 (6.8)	12 (10.3)	16 (5.5)	0.5

**Table 5:** Operative details and outcomes of the studied patients according to GFR.

Studied variables		Patients with GFR > 60	Patients with early CKD	Patients with late CKD	Patients on dialysis	*P* value
Type of surgery	Isolated CABG (*n*, %)	263 (90.1)	67 (90.5)	10 (83.3)	30 (93.8)	0.73
	CABG plus valve surgery (*n*, %)	30 (10.3)	7 (9.5)	1 (9.1)	2 (6.3)	
Cardiopulmonary bypass (minutes)		100 (79–125)	96 (75–128)	120.5 (87–153)	98 (73–110)	0.28
Aortic clamping (minutes)		63 (44.5–85)	49.5 (40–77)	62 (50–120)	48 (41–75)	0.07
Approach	Sternotomy (*n*, %)	286 (97.9)	71 (95.9)	10 (90.9)	31 (96.9)	0.28
	Minimally invasive	7 (2.4)	3 (4.1)	1 (9.1)	1 (3.1)	
Urgency of surgery	Elective (*n*, %)	282 (96.6)	71 (95.9)	11 (100)	32 (100)	0.8
	Emergent (*n*, %)	11 (3.8)	3 (4.1)	0 (0)	0 (0)	
Exploration for bleeding (*n*, %)		21 (7.2)	4 (5.4)	0 (0)	2 (6.3)	0.97
New onset AF (*n*, %)		78 (26.7)	24 (32.4)	2 (18.2)	5 (15.6)	0.3
Ventricular arrhythmias (*n*, %)		16 (5.5)	6 (8.1)	1 (9.1)	1 (3.1)	0.61
Acute kidney injury (*n*, %)		59 (20.2)	36 (48.6)	10 (90.9)	0 (0)	<0.001
New need for hemodialysis (*n*, %)		7 (2.4)	11 (14.9)	5 (45.5)	0 (0)	<0.001
Gastrointestinal bleeding (*n*, %)		11 (3.8)	8 (10.8)	1 (9.1)	0 (0)	0.044
Sternotomy wound infection (*n*, %)		38 (13)	11 (14.9)	1 (9.1)	3 (9.4)	0.9
Wound debridement (*n*, %)		23 (7.9)	11 (14.9)	1 (9.1)	2 (6.3)	0.28
ICU days		4 (3–5)	5 (3–7)	12 (4–26)	4 (3–5)	0.002
Inotrope days		1 (1-2)	2 (1–4)	4 (2–12)	2 (1-2)	<0.001
Ventilator days		1 (1-1)	1 (1-2)	4 (1–18)	1 (1-2)	0.001
Post-ICU ward days		4 (3–6)	6 (3–11)	7 (5–16)	5 (4–7)	0.006
Postoperative ECMO (*n*, %)		5 (1.7)	2 (2.7)	1 (9.1)	0 (0)	0.27
Postoperative IABP (*n*, %)		24 (8.2)	6 (8.1)	1 (9.1)	1 (3.1)	0.81
Tracheostomy (*n*, %)		6 (2.1)	4 (5.4)	2 (18.2)	0 (0)	0.02
Hospital mortality (*n*, %)		6 (2.1)	3 (4.1)	1 (9.1)	1 (3.1)	0.03
Hospital cerebrovascular stroke (*n*, %)		19 (6.5)	10 (13.5)	2 (18.2)	0 (0)	0.03
Acute ischemic stroke (*n*, %)		18 (6.2)	10 (13.5)	2 (18.2)	0 (0)	0.03
Acute intracerebral bleeding (*n*, %)		1 (0.34)	0 (0)	0 (0)	0 (0)	1
Stroke during follow-up (*n*, %)		7 (2.4)	3 (4.1)	1 (9.1)	4 (12.5)	0.028
Mortality during follow-up (*n*, %)		16 (5.5)	10 (13.5)	0 (0)	2 (6.3)	0.104

**Table 6:** Univariate analysis according to hospital mortality.

Variables		Survivors (*n* = 399, 97.3%)	Nonsurvivors (*n* = 11, 2.7%)	*P* value
Age (years)		60 (55–68)	63 (48–80)	0.39
Diabetes mellitus (*n*, %)		331 (83)	7 (63.6)	0.109
Hyperlipidemia (*n*, %)		238 (59.6)	7 (63.6)	0.92
Hypertension (*n*, %)		343 (86)	8 (72.7)	0.201
Bronchial asthma (*n*, %)		43 (10.8)	1 (9.1)	1
CKD (*n*, %)		112 (28.2)	5 (45.5)	0.01
PVD (*n*, %)		43 (10.8)	1 (9.1)	1
Preoperative AF (*n*, %)		24 (6)	2 (18.2)	0.15
Prior stroke (*n*, %)		27 (6.8)	1 (9.1)	0.54
CAS (*n*, %)		35 (8.8)	2 (18.2)	0.26
Prior MI (*n*, %)		249 (62.4)	8 (72.7)	0.75
Prior cardiac surgeries (*n*, %)		23 (5.8)	4 (36.4)	0.004
Type of surgery	Isolated CABG (*n*, %)	365 (91.5)	5 (45.5)	<0.001
	CABG plus valve surgery (*n*, %)	34 (8.5)	6 (54.5)	
Emergency surgery (*n*, %)		10 (2.5)	3 (27.3)	0.004
Cardiopulmonary bypass (minutes)		100 (77–125)	94 (89–168)	0.33
Aortic cross-clamping (minutes)		58 (43–82)	59 (45–109)	0.36
Exploration for bleeding (*n*, %)		24 (6)	3 (27.3)	0.03
New onset AF (*n*, %)		100 (25.1)	9 (81.8)	<0.001
Acute ischemic stroke (*n*, %)		23 (5.8)	7 (87.5)	<0.001
Acute kidney injury (*n*, %)		94 (23.6)	11 (100)	<0.001
New need for hemodialysis (*n*, %)		14 (3.5)	10 (90.9)	<0.001
Peak lactate (mmol/L)		5.4 (3.7–8.9)	15.9 (8.3–16.6)	<0.001
Peak troponin (ng/L)		620 (391–1170)	1250 (850–2350)	0.003

**Table 7:** Logistic multivariate regression to obtain the predictors of postoperative stroke and hospital mortality.

Variables	Odds ratio	95% CI	*P* value
Logistic regression for hospital mortality			
Emergency surgery	3.1	1.82–12.37	0.036
Acute postoperative stroke	10.289	1.823–58.08	0.008
New need for dialysis	25.617	13.78–85.47	<0.001
Prior cardiac surgery	0.369	0.016–8.46	0.53
Postoperative AF	0.403	0.017–9.382	0.57
Peak troponin level	0.9999	0.99–1.0001	0.522
Peak lactate level	0.9997	0.9992–1.00001	0.062
Logistic regression for postoperative cerebrovascular stroke			
Carotid artery stenosis	9.38	3.72–23.66	<0.001
Atrial fibrillation	2.35	1.03–5.41	0.016
CABG plus valve surgery	7.44	2.75–20.15	<0.001
HBA1c	1.43	1.071–1.91	0.016
CKD	2.33	1.06–5.17	0.036
Prior stroke	3.73	1.18–11.77	0.025

## Data Availability

The data used to support the findings of this study are available on request from the corresponding author.

## Abbreviations

CKD: Chronic kidney disease
ESRD: End-stage renal disease
AKI: Acute kidney injury
ATRIA: Anticoagulation and risk factors in atrial fibrillation
CABG: Coronary artery bypass grafting
CPB: Cardiopulmonary bypass
CAS: Carotid artery stenosis
OR: Odds ratio
HAS-BLED: Hypertension, abnormal liver/renal profile, cerebrovascular stroke, bleeding history, labile INR, elderly, drugs/alcohol concomitantly
IABP: Intra-aortic balloon pump
vWF: von Willebrand factor
ECMO: Extracorporeal membrane oxygenation.

## References

[1] Serruys P. W., Morice M. C., Kappetein A. P. (2009). SYNTAX Investigators: percutaneous coronary intervention versus coronary-artery bypass grafting for severe coronary artery disease. *New England Journal of Medicine*.

[2] Sarnak M. J., Levey A. S., Schoolwerth A. C. (2003). Kidney disease as a risk factor for development of cardiovascular disease: a statement from the American heart association councils on kidney in cardiovascular disease, high blood pressure Research, clinical cardiology, and epidemiology and prevention. *Circulation*.

[3] Anderson R. J., O’Brien M., MaWhinney S. (1999). Renal failure predisposes patients to adverse outcome after coronary artery bypass surgery: VA Cooperative Study #5. *Kidney International*.

[4] Kobayashi S. (2017). Cardiovascular events in hemodialysis patients: challenging against vascular calcification. *Annals of Vascular Diseases*.

[5] Hori D., Yamaguchi A., Adachi H. (2017). Coronary artery bypass surgery in end-stage renal disease patients. *Annals of Vascular Diseases*.

[6] Liu J. Y., Birkmeyer N. J. O., Sanders J. H. (2000). Risks of morbidity and mortality in dialysis patients undergoing coronary artery bypass surgery. *Circulation*.

[7] Yamauchi T., Miyata H., Sakaguchi T. (2012). Coronary artery bypass grafting in hemodialysis-dependent patients. *Circulation Journal*.

[8] Safaie N., Chaichi P., Habibzadeh A., Nasiri B. (2011). Postoperative outcomes in patients with chronic renal failure undergoing coronary artery bypass grafting in madani heart center: 2000-2010. *Journal of Cardiovascular and Thoracic Research*.

[9] Levey A. S., Eckardt K.-U., Tsukamoto Y. (2005). Definition and classification of chronic kidney disease: a position statement from Kidney Disease: Improving Global Outcomes (KDIGO). *Kidney International*.

[10] Levin A., Stevens P. E., Bilous R. W. (2013). Kidney Disease: Improving Global Outcomes (KDIGO) CKD Work Group. KDIGO 2012 clinical practice guideline for the evaluation and management of chronic kidney disease. *Kidney International Supplements*.

[11] Bellomo R., Ronco C., Kellum J. A., Mehta R. L., Palevsky P. (2004). Acute renal failure-definition, outcome measures, animal models, fluid therapy and information technology needs: the Second International Consensus Conference of the Acute Dialysis Quality Initiative (ADQI) Group. *Critical Care*.

[12] Cooper W. A., O’Brien S. M., Thourani V. H. (2006). Impact of renal dysfunction on outcomes of coronary artery bypass surgery. *Circulation*.

[13] Reddan D. N., Szczech L. A., Tuttle R. H. (2003). Chronic kidney disease, mortality, and treatment strategies among patients with clinically significant coronary artery disease. *Journal of the American Society of Nephrology*.

[14] Laimoud M., Maghirang M., Alanazi M. (2022). Predictors and clinical outcomes of post-coronary artery bypass grafting cerebrovascular strokes. *The Egyptian Heart Journal*.

[15] Lok C. E., Austin P. C., Wang H., Tu J. V. (2004). Impact of renal insufficiency on short-and long-term outcomes after cardiac surgery. *American Heart Journal*.

[16] Laimoud M., Qureshi R. (2020). Outcome of postcardiac surgery acute myocardial infarction and role of emergency percutaneous coronary interventions. *Cardiology Research and Practice*.

[17] Powell K. L., Smith J. M., Woods S. E., Hendy M. P., Engel A. M., Hiratzka L. F. (2004). Coronary artery bypass grafting in patients with dialysis-dependent end stage renal disease: a prospective, nested case-control study. *Journal of Cardiac Surgery*.

[18] Kan C. D., Yang Y. J. (2004). Coronary artery bypass grafting in patients with dialysis-dependent renal failure. *Texas Heart Institute Journal*.

[19] Ocak G., Rookmaaker M. B., Algra A. (2018). Chronic kidney disease and bleeding risk in patients at high cardiovascular risk: a cohort study. *Journal of Thrombosis and Haemostasis*.

[20] Linthorst G. E., Avis H. J., Levi M. (2010). Uremic thrombocytopathy is not about urea: table 1. *Journal of the American Society of Nephrology*.

[21] Fang M. C., Go A. S., Chang Y. (2011). A new risk scheme to predict warfarin-associated hemorrhage: the ATRIA (Anticoagulation and RiskFactors in Atrial Fibrillation) Study. *Journal of the American College of Cardiology*.

[22] Pisters R., Lane D. A., Nieuwlaat R., de Vos C. B., Crijns H. J., Lip G. Y. (2010). A novel user-friendly score (HAS-BLED) to assess 1-yearrisk of major bleeding in patients with atrial fibrillation: theEuro Heart Survey. *Chest*.

[23] Sarkar M., Prabhu V. (2017). Basics of cardiopulmonary bypass. *Indian Journal of Anaesthesia*.

[24] Lamy A., Devereaux P. J., Prabhakaran D. (2012). Off-pump or on-pump coronary-artery bypass grafting at 30 days. *New England Journal of Medicine*.

[25] Li X., Zhang S., Xiao F. (2020). Influence of chronic kidney disease on early clinical outcomes after off-pump coronary artery bypass grafting. *Journal of Cardiothoracic Surgery*.

[26] Ueki C., Miyata H., Motomura N. (2018). Off-pump technique reduces surgical mortality after elective coronary artery bypass grafting in patients with preoperative renal failure. *The Journal of Thoracic and Cardiovascular Surgery*.

[27] Ishigami J., Grams M. E., Chang A. R., Carrero J. J., Coresh J., Matsushita K. (2017). CKD and risk for hospitalization with infection: the atherosclerosis risk in communities (ARIC) study. *American Journal of Kidney Diseases*.

[28] Espi M., Koppe L., Fouque D., Thaunat O. (2020). Chronic kidney disease-associated immune dysfunctions: impact of protein-bound uremic retention solutes on immune cells. *Toxins*.

[29] Ikonomidis I., Makavos G., Lekakis J. (2015). Arterial stiffness and coronary artery disease. *Current Opinion in Cardiology*.

[30] Shigematsu T., Sonou T., Ohya M. (2017). Preventive strategies for vascular calcification in patients with chronic kidney disease. *Contributions to Nephrology*.

[31] Walker A. M., Schneider G., Yeaw J., Nordstrom B., Robbins S., Pettitt D. (2006). Anemia as a predictor of cardiovascular events in patients with elevated serum creatinine. *Journal of the American Society of Nephrology*.

[32] Davies G., Kingswood C., Street M. (1996). Pharmacokinetics of opioids in renal dysfunction. *Clinical Pharmacokinetics*.

[33] Jannati M., Attar A. (2019). Analgesia and sedation post-coronary artery bypass graft surgery: a review of the literature. *Therapeutics and Clinical Risk Management*.

[34] Pascarella G., Costa F., Nonnis G. (2023). Ultrasound guided parasternal block for perioperative analgesia in cardiac surgery: a prospective study. *Journal of Clinical Medicine*.

